# A new strategy for gene targeting and functional proteomics using the DT40 cell line

**DOI:** 10.1093/nar/gkt650

**Published:** 2013-07-27

**Authors:** Kinga P. Orlowska, Kamila Klosowska, Roman J. Szczesny, Dominik Cysewski, Pawel S. Krawczyk, Andrzej Dziembowski

**Affiliations:** ^1^Institute of Genetics and Biotechnology, Faculty of Biology, University of Warsaw, Pawinskiego 5a, 02-106, Warsaw, Poland and ^2^Institute of Biochemistry and Biophysics, Polish Academy of Sciences, Pawinskiego 5a, 02-106 Warsaw, Poland

## Abstract

DT40 cells derived from chicken B lymphocytes exhibit exceptionally high homologous recombination rates. Therefore, they can be used as a convenient tool and model for gene targeting experiments. However, lack of efficient cloning strategies, protein purification protocols and a well annotated protein database limits the utility of these cells for proteomic studies. Here we describe a fast and inexpensive experimental pipeline for protein localization, quantification and mass spectrometry–based interaction studies using DT40 cells. Our newly designed set of pQuant vectors and a sequence- and ligation-independent cloning (SLIC) strategy allow for simple and efficient generation of gene targeting constructs, facilitating homologous-recombination–based protein tagging on a multi-gene scale. We also report proof of principle results using the key proteins involved in RNA decay, namely EXOSC8, EXOSC9, CNOT7 and UPF1.

## INTRODUCTION

Organisms and cell lines obtained by gene targeting using homologous recombination (HR) are the most reliable model systems for studying protein function and protein–protein interactions. High-throughput gene tagging methodologies are crucial for multi-gene projects, as they allow numerous protein co-immunoprecipitation (CoIP), localization and quantification experiments to be performed in parallel using standardized protocols. For example, such high-throughput experiments done using yeast as a model organism have provided a wealth of valuable biological information, including novel information about proteins with previously unknown functions (1–6).

However, in most eukaryotic systems, gene targeting efficiency is low compared with unicellular eukaryotes, such as *Saccharomyces cerevisiae*, which is a limiting factor for high-throughput studies using this approach in *Metazoa*. Thus, alternative strategies of protein tagging in higher eukaryotic systems were developed, including the use of nucleases (ZFNs or TALENs) engineered to recognize a unique site in the genome. Design methodologies for these nucleases have improved significantly over the past years (7–9); however, there are still many limitations in the ability to choose cleavage sites and problems arising from imperfect cleavage specificity. Furthermore, designing, producing and testing the activity of each protein for every gene is labor- and cost-intensive, limiting the utility of custom nucleases in multi-gene projects. Instead of using custom nucleases for gene targeting, random integrations of artificial chromosomes (e.g. BACs) or fosmids expressing tagged proteins are often used (7,10). Nevertheless, the approach of tagging a gene at its endogenous locus has substantial advantages over such strategies that require genomic integration of large engineered DNA constructs because the former ensures that the entire pool of the protein of interest uniformly carries the desired tag, eliminating all complications due to interference from the untagged protein.

DT40 is a stable cell line, established after infection of chicken bursal lymphoblasts with an avian leukosis virus (11). Its exceptional feature is the high ratio of targeted-to-random integration of transfected DNA constructs containing regions homologous to the DT40 genome—a phenomenon firstly described in 1991 (12), which makes the DT40 cell line an excellent candidate for gene targeting experiments with high efficiency at a relatively low cost. High gene targeting efficiency can be regarded as a side effect of the elevated frequency of HR events. High level of HR is essential for diversification of the immunoglobulin repertoire by gene conversion at the light chain Ig locus in B cell precursors during their development in the bursa of Fabricius as well as in the lymphoblast-derived DT40 cell line (13). The mechanism underlying the high HR frequency in DT40 cells has not been fully understood yet. The activation-induced deaminase gene involved in B cell development has been shown to be an essential factor for gene conversion (14), and genes important for DNA damage response such as Rad52 and FANCC are also known to play an important role both in gene targeting and gene conversion (15,16). High frequency of gene targeting by HR is not absolutely unique to DT40 cells but was also reported in adeno-associated virus infected ESCs and IPSCs of human and murine origin, but these are far more difficult to culture than DT40 cells. Relatively high HR frequency was also detected in two human cell lines: a colon cancer–derived cell line HCT116 (which is an adhesive cell line) and Nalm-6 cells (a human pre-B cell line growing in suspension like DT40 but with longer doubling times). However, gene targeting in these two human cell lines was only reported in a double (positive/negative) selection system. Such a system has to be used to increase the targeted-to-random integration ratio but it makes construct cloning more difficult and raises more biosafety issues as Diphteria toxin (known to be toxic for human cells) is produced by a fraction of transfected cells. Therefore, DT40 seems to be the most suitable cell line for gene targeting experiments described so far. It should be noted, however, that some topics typical for adherent cells and absent in suspension cells cannot be studied using DT40 as a model. Similarly, data obtained on DT40, which concern processes differently organized in adherent cells, will have limited relevance to the latter. Cell adhesion and the extracellular matrix interactions surely belong to these topics. On the other hand, DT40 cells are a particularly good model e.g. for studies focused on avian B-cell development.

So far, DT40 cells have mostly been used to generate knock-out cell lines for functional analyses. Epitope tagging of endogenous proteins using DT40 cells was also reported (17–19); however, these experiments used inefficient cloning strategies and lacked reliable standardized protocols. Here, we describe a strategy for multi-gene protein interaction and localization studies, which combines sequence- and ligation-independent cloning (SLIC) (20) and gene targeting in DT40 cells. We also prove the utility and efficiency of our strategy using genes encoding proteins involved in RNA decay.

## MATERIALS AND METHODS

### Construction of the basic *pQuant* vectors

*pQuantA-hygro-amp* vector was constructed by SLIC of a 1.2-kb synthetic construct containing a *Xho*I/*Hind*III restriction site, the QuantA tag followed by a loxP L site and a downstream chicken β-actin 3′UTR into a 3.3-kb fragment of the *pSilencer 2.1-U6 hygro* (Invitrogen) vector, containing the plasmid backbone, a hygromycin resistance cassette under the control of the SV40 promoter and a loxP R site (the fragment of the pSilencer vector was amplified using primers *ADZ-KO-53* and *ADZ-KO-55;* for all oligonucleotide sequences, please refer to Supplementary Table S1).

*pQuantA-hygro-kana* vector was obtained by exchanging the amp^R^ cassette into a kanamycin resistance cassette: *the pQuantA-hygro-amp* vector was digested with *Ear*I, and the resulting 3.0-kb fragment was ligated with a 1.7-kb fragment of *pET28* containing the *ori* and kanamycin resistance cassette, obtained by polymerase chain reaction (PCR) using the *ADZ-KO-66* and *ADZ-KO-77* primers, and subsequent *Ear*I digestion.

DNA encoding EGFP was amplified by PCR from the *pEGFP-C1* plasmid (Clontech) using primers *ADZ-KO-58* and *ADZ-KO-59* and subsequently digested with *Sac*I and *Spe*I. DNA encoding the FLAG peptide was obtained by annealing the oligonucleotides *ADZ-KO-60* and *ADZ-KO-61*. To obtain *pQuantEGFP-hygro-kana* and *pQuantFLAG-hygro-kana* vectors, *pQuantA-hygro-kana* was digested with *Sac*I and *Spe*I, and the resulting 4.0-kb fragment was ligated with inserts encoding for EGFP and FLAG, respectively.

Vectors carrying puromycin or blasticidin resistance cassettes were created from hygro^R^ vectors by excision of the hygromycin resistance cassette using *Avr*II and *Sal*I and SLIC cloning of a puromycin or blasticidin resistance cassette produced by PCR on *pLoxPuro* and *pLoxBsr* plasmids (21) using the *ADZ-KO-62*, *ADZ-KO-63* and *ADZ-KO-93*, *ADZ-KO-94* primer pairs, respectively.

### SLIC of constructs for gene targeting

For each gene of interest, a pair of 2.2–2.5-kb long homology arms was designed using genomic DNA sequences from the chicken genome assembly 2.1 (22). The left arm was designed to end right before the termination codon of the open reading frame of the gene, whereas the right arm was designed to start with this termination codon.

Homology arms were produced from DT40 genomic DNA by PCR using Phusion high-fidelity DNA polymerase (Thermo Scientific) and primer pairs *ADZ-KO-1E1* and *ADZ-KO-1E2*, *ADZ-KO-1F1* and *ADZ-KO-1F2*, *ADZ-KO-2E5* and *ADZ-KO-2E6*, *ADZ-KO-125* and *ADZ-KO-126* for *EXOSC8*, *EXOSC9*, *CNOT7* and *UPF1* genes, respectively. In this reaction, 5-kb fragments containing both arms were amplified and *Not*I sites were added on primer overhangs. PCR products were purified using a Clean-Up kit (A&A Biotechnology), digested with *Not*I and circularized by self-ligation. The circular products were used as templates for a subsequent PCR reaction using Phusion high-fidelity DNA polymerase and the primer pairs *ADZ-KO-1E3* and *ADZ-KO-1E4*, *ADZ-KO-1F3* and *ADZ-KO-1F4*, *ADZ-KO-2E7* and *ADZ-KO-2E8*, *ADZ-KO-127* and *ADZ-KO-128* for *EXOSC8*, *EXOSC9*, *CNOT7* and *UPF1*, respectively. This resulted in an inversion of homology arms and addition of 30-bp ‘SLIC homology regions’ located on primer overhangs. These PCR products were gel-purified using a Gel-Out kit (A&A Biotechnology) and used as inserts for SLIC cloning into *pQuant* vectors.

SLIC was performed according to Li and Ellegde (20) with minor modifications. For each reaction, 50 ng *Hind*III/*Xho*I-digested and gel-purified *pQuantEGFP-puro-kana* or *pQuantEGFP-hygro-kana* vector and 100–300 ng 5-kb insert was mixed, and single-stranded homology regions were created by digestion with 0.5 u of T4 DNA polymerase (New England Biolabs) for 30 min at 23°C. The reaction was stopped on ice with the addition of dATP to a final concentration of 1 mM. Subsequently, vector and insert annealing was performed by incubation at 37°C for 30 min, and the reaction mixture was used to transform chemocompetent MH1 bacteria. The resulting targeting constructs (*pKO46*, *pKO50*, *pKO48*, *pKO59*, *pKO52*, *pKO66*, *pADZ450* and *pADZ525*) were confirmed by restriction digests and sequencing of exonic regions of the gene of interest. All plasmids obtained in this study are listed in Supplementary Table S2.

### Cell culture

The DT40^Cre1^ cell line (obtained from J.-M. Buerstedde) was used as the wild type (*wt*) cell line. Cells were cultured in chicken medium (CM) composed of Dulbecco's modified Eagle's medium (Gibco), 10% fetal bovine serum (Gibco), 1% chicken serum (Sigma), penicillin/streptomycin, 0.1 mM β-mercaptoethanol (Sigma) at 37°C with 5% CO_2_.

### Generation of stable cell lines expressing tagged proteins from endogenous loci

DNA for gene targeting in DT40 cells was purified from 100 ml bacteria culture using Plasmid Midi kit (A&A Biotechnology). Forty microgram of plasmid DNA was linearized with 50 U of *Not*I for 16 h at 37°C and purified by ethanol precipitation.

Stable transfection of DT40 cells was carried out according to Saribasak and Arakawa (23) with minor modifications. DT40 cells were grown in CM medium to a density of 0.5–1.0 × 10^6^ cells/ml. A total of 10^7^ cells were pelleted at 500*g* for 5 min at 23°C and resuspended in 800 μl CM in a precooled BioRad 4 mm electroporation cuvette. After the addition of the linearized targeting DNA, electroporation was done using BioRad Gene Pulser at 700 V and 25 μF. After electroporation, cells were mixed with 10 ml CM and cultured for 12–24 h. Selective antibiotic was then added (puromycin to a final concentration of 0.5 μg/ml or hygromycin to a final concentration of 2.5 mg/ml) and cells were placed in 96-well plates (200 μl/well). After 7–14 days, cells from wells containing a single colony were expanded on 24-well plates. Genomic DNA was isolated using Genomic Mini kit (A&A Biotechnology). Correctly targeted clones were identified by PCR (Supplementary Figure S1). Homozygous cell lines, obtained after two rounds of successful transfection, were grown in the presence of 5 μM 4-hydroxytamoxifen for 48 h to activate Cre recombinase, and subcloned. After 5–7 days of colony growth, the loss of puromycin- and hygromycin-resistance was tested to confirm Cre-mediated recombination of loxP-R and loxP-L sequences.

### Flow cytometry analysis

To compare protein levels, 5 × 10^5^ cells were pelleted at 500*g* for 3 min, washed with phosphate buffered saline (PBS), pelleted again and resuspended in 0.5 ml PBS. Fluorescence was measured using a FACScalibur instrument (BD Biosciences). Cyflogic software (CyFlo Ltd.) was used for FACS data analysis. Forward scatter and side scatter measurements were used to identify living cells for the calculation of mean fluorescence intensity. To measure protein levels during cell cycle, 1 × 10^6^ cells were pelleted at 500*g* for 3 min, resuspended in 50 µl of ice-cold PBS and fixed by adding 1 ml of ice-cold 70% ethanol. After 1 h of incubation at 4°C, cells were centrifuged, harvested in PBS solution containing RNase A (50 µg/ml, Sigma) and propidium iodide (50 µg/ml, Sigma) and incubated for 30 min at room temperature before flow cytometry analysis. A manual gating was performed using Cyflogic Software. Doublets were removed based on FL2-A and FL-2W analysis.

### Detection of EGFP-tagged proteins by western blotting

Cell extracts for western analysis were obtained by sonication using Bioruptor (Diagenode) for five cycles of 30s on/30s off with ultrasonic wave output power 200 W in RIPA buffer with protease inhibitors. Western blotting was performed according to standard procedures, using mouse monoclonal anti-GFP antibody (sc-9996, Santa Cruz Biotechnology) as the primary antibody (1:1000–1:500) and peroxidase conjugated goat anti-mouse antibody (401215, Calbiochem) as the secondary antibody. Immun-Star WesternC Chemiluminescence Kit (Bio-Rad) was used for detection of the chemiluminescence signal.

### Live cell imaging

Stable cell lines expressing EGFP-tagged proteins of interest were cultured for 24 h in 400 μl CM on an eight-well Lab Tek II chamber slide (Nunc) coated with poly-lysine. Hoechst 33342 stain (1 μg/mL) was added to the wells 30 min before microscopy. Live cell imaging was performed using an Olympus FV10i confocal microscope at 37°C, with a 60 × 1.2 water-immersion objective. Images were processed using Olympus Fluoview Viewer software.

### Polyribosome profile analysis

Polyribosome profiling was done according to Esposito *et al.* (24) with some modifications. Cells were grown in CM to a density of 0.3–0.7 million/ml. For each tagged protein, profiling was performed in two variants: polyribosome profiling of cells after cycloheximide treatment (100 μg/ml, 30 min under normal cell culture conditions) and a run-off profile (without cycloheximide treatment).

Continuous sucrose gradients containing 50 mM Tris–HCl (pH 7.4), 50 mM NaCl, 1 mM DTT, 7–47% sucrose and 12 mM MgCl_2_ (for profiles after cycloheximide treatment) were prepared using an ÄKTA FPLC machine and stored at 4°C.

Cells were pelleted at 4°C, resuspended in 1 ml cold lysis buffer (20 mM HEPES, pH 7.4, 200 mM KCl, 1% Triton X-100 (reduced), 2 mM DTT and for cycloheximide treatment, with 100 μg/ml cycloheximide, 15 mM MgCl_2_ and 1 mg/ml heparin). Cells lysis was done by passing the cell suspension through a cold 30-gauge syringe needle twice. The cell lysate was then centrifuged at 16 100*g* for 5 min at 4°C. Supernatant was collected and 10 OD_260nm_ units were transferred onto 10 ml sucrose gradients, covered with 600 μl mineral oil and centrifuged at 261 000*g* for 120 min at 4°C. Gradient fractions were collected using an ÄKTA FPLC machine, precipitated with PRM reagent, resuspended in 6 M urea, diluted with 2× Laemmli buffer and used for western blotting using 1:500 anti-GFP antibodies.

### Protein precipitation using PRM reagent

Protein precipitation was done according to Aguilar *et al.* (25). PRM reagent (0.25 volume of 0.05 mM pyrogallol red, 0.16 mM sodium molybdate, 1 mM sodium oxalate, 50 mM succinic acid, pH 2.5) was added to one volume of protein containing sample and incubated at 23°C for 20 min. The precipitate was pelleted by centrifugation at 16 100*g* for 15 min at 23°C and the supernatant was discarded.

### Purification of protein complexes by CoIP

For each CoIP, 300 million cells were pelleted, washed with PBS and resuspended in 3 ml lysis buffer (10 mM Tris/HCl, 100 mM NaCl, 0.5% Triton X-100, 1 mM PMSF, 2 μM pepstatin A, 2 μg/ml chymostatin, 0.6 μM leupeptin, 2 mM benzamidine, 1 mM DTT and additionally, 2.5 mM MgCl_2_ for bait protein UPF1) and sonicated using Bioruptor (15 cycles of 30 s on/30 s off at 250 W). The lysate was centrifuged at 3200*g* (15 min, 4°C) and the supernatant was collected. The remaining pellet was resuspended in a new portion of lysis buffer, sonicated, centrifuged as above and pooled with the former supernatant. The resulting cell lysate was digested with RNase A (0.1 mg/ml, 15 min, 4°C). After digestion, the cell extract was centrifuged at 3200*g* (15 min, 4°C).

Anti-GFP bead suspension (50 μl, GFP-Trap from Chromotek) was washed with IPP100 buffer, mixed with the cell extract and rotated for 1 h at 8°C in Polyprep columns (BioRad). The flow-through was discarded, proteins bound to beads were washed with 10 ml cold IPP100 buffer (10 mM Tris/HCl, 100 mM NaCl, 0.1% Triton X-100, optionally 2.5 mM MgCl_2_) and subsequently with 10 ml cold TEV cleavage buffer (10 mM Tris/HCl, 100 mM NaCl, 0.5 mM EDTA, 1 mM DTT, optionally 2.5 mM MgCl_2_). Proteins were eluted from beads by enzymatic digestion using 5 μg TEV protease in 200 μl TEV cleavage buffer, carried out in the column for 2 h at 23°C. After digestion, the eluate was collected by gravity flow and additionally with two portions of 250 μl TEV cleavage buffer. The eluate was divided in two portions, both of which were precipitated with PRM reagent. One pellet was used for electrophoresis on a 4–12% NuPAGE gel (Invitrogen) and the other was subjected to tryptic digestion followed by LC-MS analysis.

### Tryptic digestion and mass spectrometry analysis

Tryptic digestion of the protein pellet in NH_4_HCO_3_ buffer (with no chaotropic agents), peptide reduction and alkylation were carried out according to Kim *et al.* (26). The protein pellet was resuspended in 1 ml 100 mM NH_4_HCO_3_ (pH 8.0) by sonication (three cycles, 20 s on/30 s off, 250 W, 4°C). Tryptic digestion was performed for 16 h at 37°C using 0.5 μg sequencing-grade trypsin (Promega). Peptide reduction was done by incubation of the peptide mixture with 10 mM DTT (30 min, 50°C), followed by alkylation using 30 mM iodoacetamide (30 min, 23°C) in the dark and additional digestion with 0.25 μg trypsin (3 h, 37°C). The resulting peptide mixture was lyophilized and resuspended in 0.1% TFA. Then, 0.5 pmol of isotopically labeled Quant peptide LAADITSLY[Lys(^13^C_6_;^15^N_2_)] was added.

MS analysis was performed by LC-MS in the Laboratory of Mass Spectrometry (IBB PAS, Warsaw) using a nanoAcquity UPLC system (Waters) coupled to an LTQ-Orbitrap Velos mass spectrometer (Thermo Scientific). Peptides were separated by a 160-min linear gradient of 95% solution A (0.1% formic acid) to 35% solution B (acetonitrile and 0.1% formic acid). The mass spectrometer was operated in the data-dependent MS-MS^2^ mode, acquiring data in the m/z range of 300–2000.

MS data was analyzed using Andromeda/MaxQuant software (27,28). The database used for protein identification consisted of (i) UniProt (*Gallus gallus*, 23 395 sequences; human, 70 136 sequences), (ii) a set of 3493 protein sequences resulting from mapping of DT40 transcriptome deep sequencing data (obtained by our group) onto UniProt, (iii) sequences of chicken homologues of known interactors of the proteins studied, deduced from EST data for those proteins missing from aforementioned databases and (iv) TEV protease and Quant peptide.

Quantification of the isotopically labeled Quant peptide and bait proteins was manually verified by analysis of peptide maps obtained from raw LC-MS data using MSConvert and MSparky software (http://proteom.ibb.waw.pl/).

### Transcriptome-derived protein database

Total RNA was isolated using a standard protocol, and rRNA was removed using Ribo-Zero™ Nonmagnetic Kit (human/mouse/rat, Epicentre). A standard Illumina pair-end library was prepared using Truseq RNA Sample preparation kit v.2, and sequenced on Illumina HiSeq2000. The ∼22 Gb of sequencing data obtained were used for transcriptome *de novo* assembly using Trinity software (29). A Perl script available in Trinity (transcripts_to_best_scoring_ORFs.pl) was used to identify coding sequences, yielding 145 725 sequences, which were translated and subjected to a BLASTP search against human and chicken proteomes (UniProt), with e-value cut-off set to 1e-10. Proteins were annotated using an in-house Perl script, which assigns a protein name based on the BLAST hit with the lowest e-value, separately for human and chicken hits. The longest representative sequences from each transcript group identified by Trinity were included in the final data set, which consisted of 3493 sequences.

## RESULTS

### Basic vectors and high-throughput generation of gene targeting constructs using SLIC

**Figure 1.**
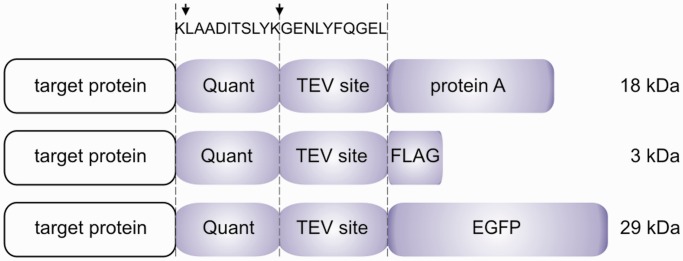
C-terminal tags designed for tagging ORFs by gene targeting in DT40 cells. Each tag consists of a quantification peptide (Quant), a TEV protease cleavage site and a protein A, FLAG or EGFP tag. Sequences of the Quant peptide and the TEV protease recognized site are shown. Arrows indicate sites of tryptic cleavage.

Our aim was to create a framework for proteomic studies using epitope tagging of native proteins. We decided to use gene targeting for integration of genetic constructs encoding one of three C-terminal protein tags at the endogenous locus of genes of interest (Figure 1). Each tag consisted of a Quant peptide, which is a modified version of the SH-quant peptide designed for protein abundance measurements by MS (30), and a TEV protease cleavage site followed by protein A, FLAG epitope or EGFP.

The EGFP containing tag seems to be the most versatile, as it allows for a range of *in vivo* analyses including live cell fluorescence microscopy and flow cytometry but the two other tags may be more suitable for particular applications. Because vectors currently used for gene targeting in DT40 cells hamper an easy and high-scale creation of required DNA constructs, we designed a novel plasmid construction strategy that meets these criteria.

Importantly, the standard gene targeting approach in DT40 cells (31) requires many restriction-site–dependent cloning steps to create a targeting construct, including separate cloning steps for each homology arm. To overcome these limitations, we reduced the cloning procedure to a single step of SLIC (20), which is a straightforward method using HR *in vitro*. In SLIC, the insert ends are designed to possess ∼30 bp identity to ends of the linearized vector of choice. Exonucleolytic activity of T4 DNA polymerase is used to reveal single stranded regions complementary between vector and insert, which are then annealed *in vitro*. Gaps in the resulting recombinant molecules are eventually filled by the DNA repair system of the bacteria used for cloning. The implementation of SLIC method eliminated the need of any unique restriction sites at the ends of the inserts, greatly simplifying the cloning procedure.

We constructed a set of basic vectors containing a tag, a downstream chicken β-actin 3′UTR, the vector backbone with selection markers for *Escherichia coli* (kanamycin or ampicillin) and resistance cassettes (puromycin, hygromycin or blasticidin) for selection of stable DT40 integrants. Importantly, the genes used for selection in DT40 are under the control of the SV40 promoter instead of the chicken β-actin promoter. Although the latter is widely used in DT40 cells, it is known to impede PCR verification of integration sites, probably due to a high GC content (21), therefore we redesigned the resistance cassettes.

The β-actin 3′UTR, resistance cassettes, and the vector backbone are flanked by LoxP sites, which allow for their removal by the Cre-mediated DNA recombination in DT40 cell lines expressing Cre recombinase. Nuclear localization and activity of Cre can be controlled by 4-hydroxytamoxifen. After induction of LoxP-directed recombination, the target gene will have a natural genomic environment with only tag sequence inserted into its coding sequence. This allows for natural expression levels. The basic vectors we designed and created (*pKO1-pKO10*, Supplementary Table S2), which are available through ADDGENE (http://www.addgene.org/), serve as backbones for the generation of gene targeting constructs.

**Figure 2.**
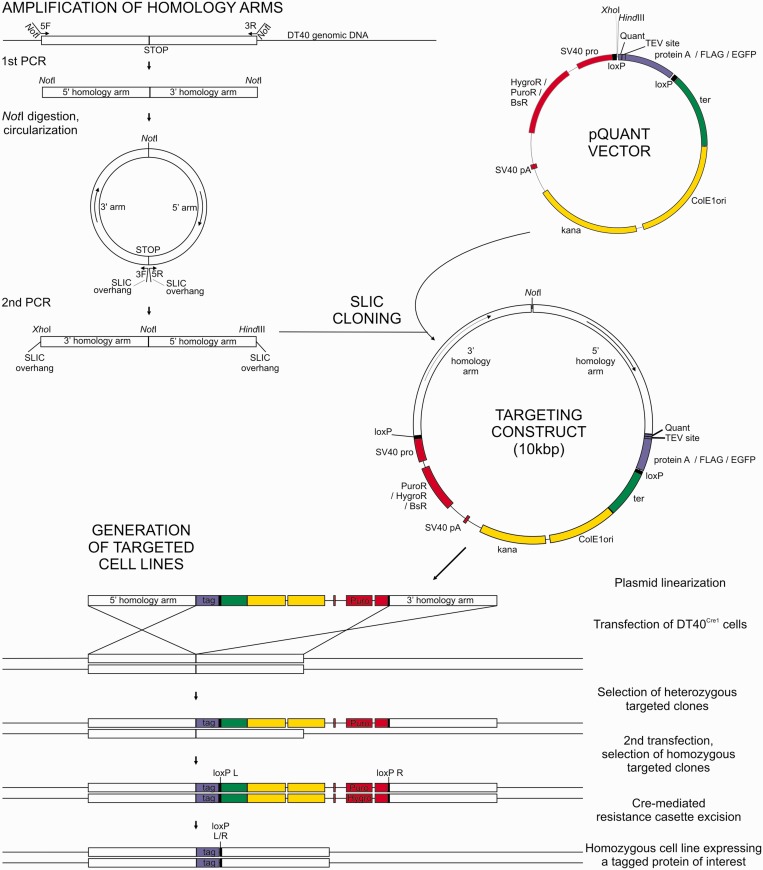
Cloning of targeting constructs and cell line generation strategy. A SLIC method was implemented to enable high-throughput cloning of constructs for protein tagging. Inserts containing gene-specific ∼2.5 kb homology arms were created using DT40 genomic DNA as a template by two subsequent PCR reactions and recombined with *Hind*III/*Xho*I-digested vector. The gene targeting constructs were used for DT40 transfection after linearization with a single-cutting restriction enzyme (e.g. *Not*I, *Asc*I, *Sna*BI, *Mlu*I, *Bgl*II). A set of vectors was designed, which enable homozygous C-terminal tagging with protein A, FLAG or EGFP and recycling of selection markers by Cre-mediated excision of resistance cassettes.

The outline of the cloning and cell line generation strategy is illustrated in Figure 2. The targeting DNA is ∼10 kb in size. The ∼5-kb vector, which is designed to be entirely integrated into the DT40 genome, is flanked by ∼2.5-kb DNA fragments, called homology arms, identical to the genomic sequences surrounding the intended integration site, which enable HR events at a frequency routinely exceeding 10% of stable integrants. First, a single genomic DNA fragment containing both homology arms is PCR-amplified. Primers (5F and 3R, Figure 2) possess overhangs with restriction sites, allowing PCR product circularization. After self-ligation, the PCR product serves as a template for reamplification using internal primers complementary to the insertion site (3F and 5R, Figure 2). The second PCR reaction reverses the homology arms and makes them SLIC-competent, as 30-bp fragments complementary to vector ends are added to inserts on primer overhangs.

### Generation of stable cell lines expressing tagged proteins

Using our strategy, stable DT40 cell line generation is a relatively rapid process. Targeting DNA is delivered into cells (in our case, DT40^Cre1^ cells) by electroporation. After 1–2 weeks of selection, colonies are screened for a HR event.

For most genes, two rounds of electroporation, selection and testing are required to obtain a homozygous cell line. To target genes located on the sex chromosome (Z), hemizygotes can be obtained after a single transfection. On the other hand, due to trisomy of chromosome 2 and microchromosome 24 in DT40 cells, three transfections are required to successfully target genes on these chromosomes. To facilitate multiple transfection and selection cycles, we constructed three vectors with different selection markers (Puro^R^/Hygro^R^/Bs^R^, Supplementary Table S2). Because all resistance cassettes can be excised by the Cre recombinase, selection markers can be recycled, which enables further modifications of the targeted cells.

To verify the functionality of our vectors and design strategy, we targeted four genes whose products are involved in RNA metabolism. We chose EXOSC8, EXOSC9, CNOT7 and UPF1 proteins because of their importance for RNA metabolism and some prior knowledge about their functional interactors. Selected genes were modified to express proteins fused to the QuantEGFP tag at the C-terminus.

The QuantEGFP tag allowed us to use flow cytometry for initial screening of clones obtained after stable transfection, limiting the number of clones that had to be tested by PCR. It should be noted that although targeted clones tend to exhibit higher fluorescence levels than non-targeted clones, if the abundance of the tagged protein is low, fluorescence levels can be similar to those of wild type cells. The exchange of the chicken β-actin promoter in the selection marker for the SV40 promoter enabled PCR verification of homozygous cell lines using gene-specific primers (i.e. products containing the whole resistance cassette were amplified to detect a targeted integration event, Supplementary Figure S1). Hence, our design allowed us to overcome the technical difficulties with PCR verification reported previously in the DT40 research community (21). After confirming homozygosity of the established cell lines (Supplementary Figure S1B–E), resistance cassettes were excised by transient induction of the Cre recombinase using 4-hydroxytamoxifen. The expression of tagged proteins of interest in the finalized cell lines was confirmed by western blotting (Supplementary Figure S2).

### Flow cytometry and fluorescent microscopy–based assays for comparative assessment of protein abundance and localization in living cells

**Figure 3.**
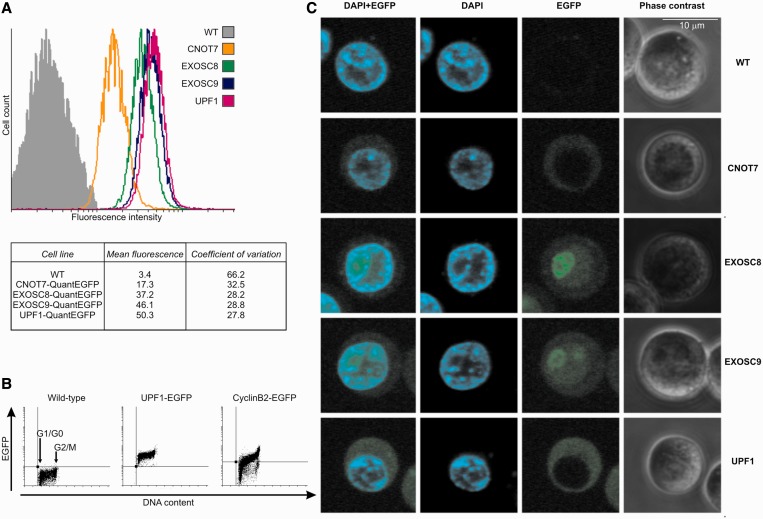
Knock-in of the QuantEGFP tag enables reliable analysis of protein localization and estimation of protein abundance in DT40 cells. (**A**) Analysis of cell population fluorescence by flow cytometry. (**B**) Analysis of the level of EGFP-tagged proteins during cell cycle progression. Parental cell line (WT) and its derivatives, homozygous UPF1-QuantEGFP and heterozygous cyclinB2-QuantEGFP, were analyzed. Cells were fixed, labeled with propidium iodide (to measure DNA content) and fluorescence was measured using flow cytometry. Points below the horizontal line represent cells negative for QuantEGFP. (**C**) Live cell fluorescence imaging of untransfected DT40 cells and stable cell lines expressing QuantEGFP-tagged CNOT7, EXOSC8, EXOSC9 or UPF1 protein.

DT40 cells grow in suspension, which makes them particularly suitable for flow cytometry–based studies. Flow cytometry can be used to estimate and/or compare protein abundance in living homozygous cell lines expressing QuantEGFP-tagged proteins of interest, providing that fluorescent properties of EGFP remain unaffected by its fusion to other proteins. Using this approach, we compared abundance of the tagged proteins in the established cell lines by calculating mean fluorescence values (Figure 3A). Our analysis indicated that UPF1 is the most abundant among the proteins measured, whereas CNOT7 is the least abundant one. Components of the exosome complex (EXOSC8 and EXOSC9) are present in the cell at similar levels, which is expected as they have a 1:1 stoichiometry within the complex. Importantly, our findings for these proteins are in agreement with data obtained for their yeast homologs. For example, in a high-throughput study by Ghaemmaghami *et al.* (6), concentrations of the yeast homologs were quantified to be 3180 copies of EXOSC8, 4800 of EXOSC9, 1520 of CNOT7 and 6090 copies of UPF1 per cell.

Moreover, compatibility of DT40 cells with flow cytometry methods allows more detailed studies. For example, changes in protein levels can be easily measured during cell cycle progression. To demonstrate this, we measured the level of UPF1 protein and cyclinB2 during the cell cycle. The level of the latter protein is known to be cell-cycle dependent and reaches its maximum in G2/M phase (32). To the best of our knowledge, the levels of UPF1 during the cell cycle have not been reported yet. We found that UPF1 levels remained constant during the cell cycle, whereas the level of cyclinB2 changed, achieving maximum in G2/M (Figure 3B).

DT40 cells are also suitable for *in vivo* microscopy, as they can easily attach to poly-lysine–coated slides. Taking advantage of this, we investigated the *in vivo* localization of EGFP-tagged EXOSC8, EXOSC9, CNOT7 and UPF1 proteins expressed at endogenous levels using live-cell imaging (Figure 3C), which has never been done before for these proteins. We found that the exosome core subunits EXOSC8 and EXOSC9 were localized to both the nucleus and the cytoplasm, with a clear enrichment in the nucleolus, which is consistent with previous localization analysis of these proteins both in unicellular and higher eukaryotes (33).

For CNOT7 (CAF1) protein, many experiments performed in yeast showed a cytoplasmic localization, although the Ccr4–Not complex was initially described as a transcription regulator (34) and was therefore believed to be a nuclear complex (1). Similarly, studies in mouse NIH3T3 cells (35) indicated a mainly cytoplasmic localization. We detected CNOT7 exclusively in the cytoplasm, which is consistent with its known function in mRNA deadenylation.

In the case of UPF1, conflicting localization data existed, reflecting a need for a reliable localization method, alternative to immunofluorescence (IF). Some IF studies detected UPF1 exclusively or mainly in the cytoplasm in various human cell lines (36–38). However, Human Protein Atlas project, which also relies on IF, detected UPF1 mainly in the nucleus in other human cell lines, a result that is inconsistent with the major known function of the protein. In yeast, UPF1 localizes primarily to the cytoplasm and shuttles in and out of the nucleus. We found that the EGFP-tagged UPF1 localized to the cytoplasm, which is consistent with the known role of UPF1 in triggering degradation of nonsense mRNA during translation.

### Protein–protein interaction and polysome association analysis

As our method of tagging proteins using HR interferes minimally with normal biology, it is particularly suitable for biochemical analyses where unperturbed physiology is of particular importance. For example, our method is suitable for polyribosome profiling, which uses ultracentrifugation of cell lysates in sucrose gradients to separate distinct fractions of ribonucleoproteins into ribosomal subunits, monosomes and polyribosomes based on differences in sedimentation rates.

**Figure 4.**
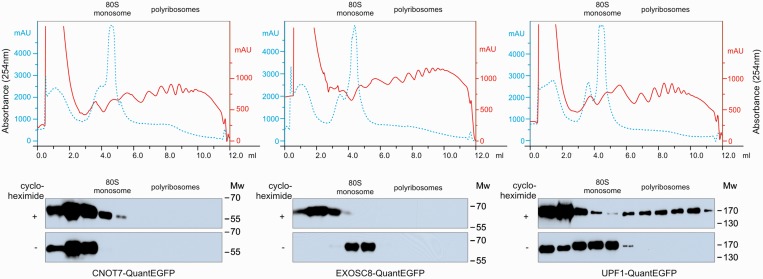
Analysis of associations between CNOT7, EXOSC8, UPF1 and the translational machinery by polyribosome profiling. Extracts from cells treated with (red solid line) and without (blue dotted line) cycloheximide were fractionated by ultracentrifugation in 7–47% sucrose gradients. Tagged proteins were detected by western blotting using anti-GFP antibodies.

We developed a protocol for polyribosome profiling using three established DT40 cell lines. We used the cell line expressing QuantEGFP-tagged UPF1 as a positive control, as UPF1 is known to associate with the translational machinery. In agreement with previous reports (39), we found that UPF1-QuantEGFP co-sedimented with polysomes (Figure 4). Neither EXOSC8 nor CNOT7 were present in the polysomal fraction, suggesting that EXOSC8- and CNOT7-dependent pathways are not associated with actively translating ribosomes. Our results indicate that our polyribosome profiling protocol using DT40 cell lines expressing tagged proteins is effective and practical.

**Figure 5.**
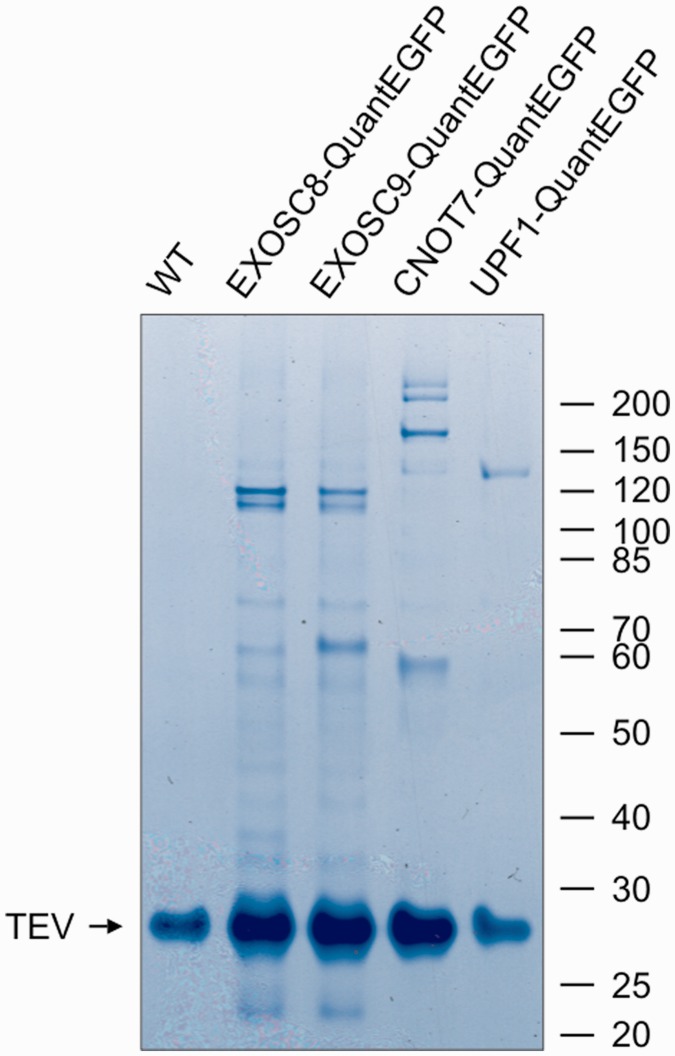
CoIP of QuantEGFP-tagged EXOSC8, EXOSC9, CNOT7 and UPF1 proteins (lanes 2, 3, 4, 5, respectively) and control purification using WT cells (lane 1). The band corresponding to TEV protease is indicated with an arrow.

All tags designed by us contain epitopes that can be used for CoIP studies. In this study, we used the QuantEGFP tag due to the availability of a high-quality anti-GFP affinity resin (GFP-Trap, Chromotek). Homozygous DT40 cells expressing QuantEGFP-tagged EXOSC8, EXOSC9, CNOT7 and UPF1 proteins were used to analyze protein–protein interactions. Protein complexes were purified by single-step affinity chromatography followed by TEV protease-mediated elution. Each bait protein was analyzed in duplicate and with control purifications performed in parallel using parental DT40^Cre1^ cells. Eluates were analyzed both by SDS-PAGE (Figure 5) and in-solution tryptic digestion followed by LC-MS. Protein identification was done by an Andromeda search against several protein sequence databases. Initially, we were not able to identifiy some putative orthologs of components of the exosome and Ccr4–Not complexes. Subsequent bioinformatics analysis revealed that these putative orthologs are absent in the UniProt database for *G.gallus* owing to incompleteness of the chicken genome assembly (especially assembly 2.1, which was used for automatic prediction of numerous chicken protein sequences present in UniProt) and poor genome annotation.

To complete the database, we decided to deep sequence the rRNA-depleted transcriptome of parental DT40^Cre1^ cells. As a result, a dedicated protein sequence database was created, which contained both chicken proteins present in UniProt and those identified through our sequencing, assembly and annotation efforts. We identified 3493 protein sequences, 384 of which did not have any homologs in the chicken UniProt database (data are accessible on the Web site of our lab http://adz.ibb.waw.pl/DT40/ and have been deposited in the NCBI SRA databank: BioProject PRJNA203279). Some known interactors (for instance, the CNOT3 subunit of the Ccr4–Not complex) were still not detected by the search engine using this database; however, they could be identified with the addition of the human UniProt proteome to the database. Some proteins (for instance three exosome components) could not be identified using the human UniProt data set owing to too little protein similarity; however, they could be identified with the addition of the corresponding chicken or turkey EST sequences to the database. All these data indicate that the annotation of chicken/DT40 genome and proteome still need to be improved what we are trying to achieve by further DT40 transcriptome and genome sequencing.

**Figure 6.**
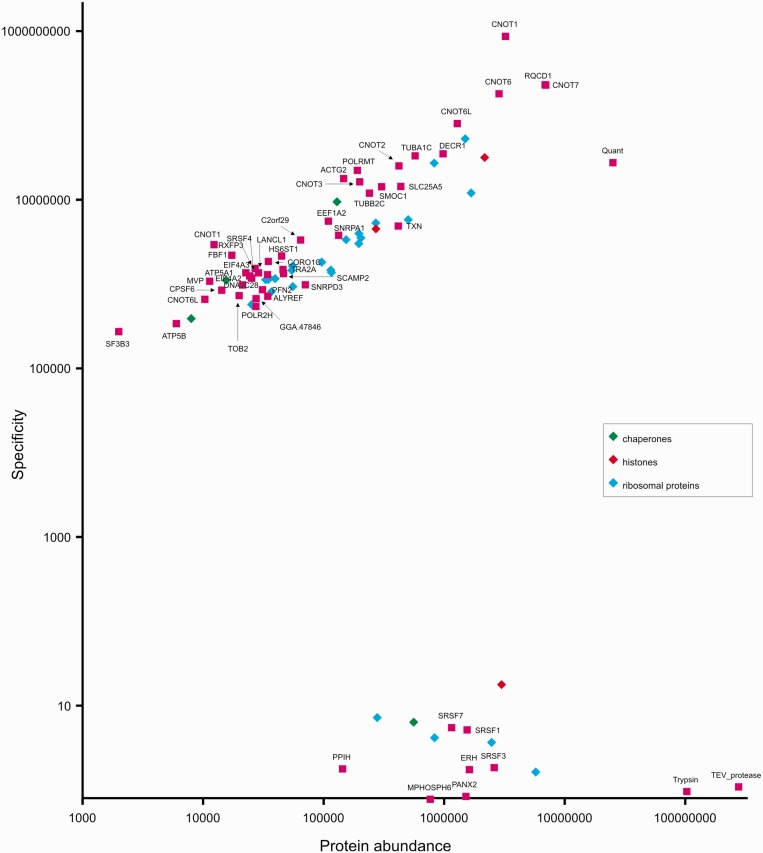
Semiquantitative analysis of CoIP results using QuantEGFP-tagged CNOT7 protein as bait. Protein abundance was defined as the mean signal intensity calculated by MaxQuant software for a protein (mean value from two replicates) divided by its molecular weight. Specificity was defined as the ratio of protein signal intensity measured in the bait purification to background level (which is the protein signal intensity in the negative control purification; background level was arbitrarily set to 1 for proteins not detected in the negative control).

MaxQuant software was used to match peptides between MS runs (to deal with the possible problem of insufficient sequencing during LC-MS runs) and to rank the identified proteins according to MS signal intensity. Protein abundance was defined as the mean signal intensity calculated by MaxQuant software for two replicates divided by the molecular mass of the protein. Specificity was calculated as the ratio of protein signal intensity measured in the bait purification to intensity determined for the negative control purification (parental DT40 cells). Calculated protein abundance versus purification specificity was plotted, as shown in Figure 6 and Supplementary Figures S3–S5.

Our CoIP analysis indicated that EXOSC8 and EXOSC9 interacted with the 9-subunit exosome core, together with its nuclear and cytoplasmic catalytic subunits, including RRP6, DIS3, DIS3L (Supplementary Figures S3 and S4). Moreover, several exosome cofactors and other proteins previously shown to co-purify with this complex from human cells (40) were also detected in eluates, such as SKIV2L2, Serine/arginine-rich splicing factors, MPP6 and numerous ribosomal proteins.

CoIP with CNOT7 resulted in identification of homologs of all known core components of the human Ccr4–Not complex (Figure 6), except for CNOT8, which is known to be a component mutually exclusive to CNOT7. Although sequences of CNOT10 and TAB182 proteins, which have been recently shown to form a new module of the Ccr4–Not complex both in the fly and in human (41), were present in our protein database, we did not detect them in our purification. This may indicate that, in vertebrates, these two proteins are associated with CNOT8-containing Ccr4–Not complexes, and not with complexes containing CNOT7. We also identified TOB2 in our immunoprecipitation with CNOT7, which was expected, as it is a homolog of a known nuclear interactor of CNOT7.

CoIP using tagged UPF1 protein was highly sensitive to purification conditions. Only some of previously known interactors, such as UPF2, UPF3A, UPF3B, SMG1, eRF1, eRF3 and PABP1 (37,42–44), were identified, and the results varied significantly among several CoIP trials. For example, we found that the presence of Mg^2+^ ions in lysis and washing buffers was essential for the stability of the Upf1–Upf2 interaction. Some of the interactions might not have been detected owing to their transient nature, as these interactors have different subcellular localizations. For instance, Upf3 is mainly a nuclear protein, Upf2 is perinuclear, whereas Upf1 is mainly cytoplasmic. In sum, our CoIP experiments demonstrated that DT40 cells stably expressing tagged proteins are useful for proteomic studies.

### Protein abundance analysis

Knowledge of protein copy number and description of protein complex stoichiometry is important for understanding cellular processes at the molecular level. To be able to measure absolute protein abundance, we designed an 11-residue peptide (KLAADITSLYK) at the C-terminus of the bait protein, which remains after cleavage with TEV protease. Therefore, after tryptic digestion, a 10-residue Quant peptide (LAADITSLYK) is present in 1:1 stoichiometry with the bait protein (Figure 1). The addition of a ‘heavy’ version of this peptide, containing a lysine residue labeled with stable isotopes ^13^C_6_,^15^N_2_ (which results in a mass increase of 8 Da), enables the absolute quantification of the bait protein by mass spectrometry. We tested the dynamic range of the quantification on an Orbitrap Velos mass spectrometer (Thermo Scientific) in the absence of other peptides and obtained a linear relation between peak volume and Quant peptide concentration in the range of 250 pM to 250 nM (data not shown). Using this approach, it is theoretically possible to calculate bait protein copy number per cell. With the help of semiquantitative data provided by MaxQuant, protein complex stoichiometry can be determined, providing a valuable tool for protein–protein interaction studies.

Using this strategy, we detected 69 ± 23 fmol CNOT7, 20 ± 8 fmol EXOSC8, 134 ± 9 fmol EXOSC9 and 194 ± 36 fmol UPF1 in eluates from 4 × 10^7^ DT40 cells. The major source for variation in quantification resulted from variability of cell and nuclear lysis efficiency during extract preparation. Assuming a 50% CoIP yield, our measurements indicate that there are 2000, 600, 4000 and 6000 copies of CNOT7, EXOSC8, EXOSC9 and UPF1 proteins, respectively, per DT40 cell. These data correlate well with flow cytometry analysis results for CNOT7 and UPF1 proteins. However, for exosome subunits, a surprisingly high EXOSC9/EXOSC8 ratio was obtained, which did not correlate with protein abundance estimates calculated by MaxQuant software (Supplementary Table S3). Western blotting (Supplementary Figure S2) and manual inspection of peptide signal intensities (data not shown) revealed that tagged EXOSC9 undergoes degradation close to its endogenous C-terminus. As a result of this degradation, the Quant peptide signal intensity does not reflect the true concentration of functional EXOSC9 protein in the cell. Therefore, we suggest manual inspection of peptide signal intensities as a quality control measure for this application of our DT40-based protein expression system.

## DISCUSSION

We developed a pipeline for fast and simple stable vertebrate cell line development. Our approach makes experiments with endogenously tagged proteins in vertebrate cells easy and efficient. The use of a SLIC cloning method enables easy construct design and could possibly be further simplified by implementation of the recently developed ‘SLiCE’ cloning method (45).

The overall methodology we developed has several advantages over other tagging systems. One method of choice involves BAC integration into the genome of human cells. However, this requires RNAi-mediated silencing of the endogenous gene, which is time-consuming. Without silencing, the BAC approach results in altered expression levels due to the introduction of an additional gene copy, and cannot eliminate the presence of native untagged protein. Moreover, random integration into the genome can cause large clone-to-clone variability of protein expression levels. Thus, the BAC approach may require screening for a cell line expressing tagged protein at the same level as the endogenous protein. Another method is the construction of knock-in mice or murine or human ES or IPS cells, which is far more time-consuming and expensive than our strategy. Moreover, DT40 cells are among the fastest growing vertebrate cell lines, with a doubling time of ∼11 h. The fact that they grow in suspension allows for easy scaling up as well as the production of large quantities of starting material. Finally, high translational activity and abundant polysomes in these cells may be useful for studying proteins involved in cytoplasmic RNA metabolism.

One may worry that the use of every nonmammalian model system (and even the use of some human-derived stable cell lines like HeLa cells) may raise doubts about the relevance of results to human biology. The system we present is not too much more difficult to use than baker's yeast but surely more relevant to mammalian systems. As examples supporting this assumption, we presented proteomic results obtained using four DT40 cell lines expressing C-terminally tagged proteins: EXOSC8, EXOSC9, CNOT7 and UPF1, which are involved in RNA decay (Results, Supplementary Table S3 and Supplementary Figures S3–S6). These data are generally consistent with the current knowledge about these proteins obtained using human cell lines.

Most of experiments presented in this study use a relatively large C-terminal tag containing GFP, which may have an effect on the target protein function. Importantly, in a high-throughput study by Gavin *et al.* (1) conducted in haploid yeast, it was shown that 82% of essential proteins remained functional after C-terminal tagging with the classical TAP tag (of 20 kDa size). These data strongly suggest that the vast majority of proteins can be C-terminally tagged without loss of protein function in higher eukaryotic cells. Indeed, a recent article of Stadler *et al.* (46) showed high correlation for protein localization in mammalian cells between exogenously expressed fluorescent-protein tagged proteins and their natural counterparts visualized by IF. Notably, C-terminal fusion proteins, also applied in our strategy, turned out to be more biologically relevant than N-terminal fusions. In our study, we designed three tags of 18 kDa, 3 kDa and 29 kDa size, and the experimental results presented here were obtained using the largest one. In the case of the four studied proteins, the large C-terminal tag does not preclude protein complex formation, nor does it seem to influence the protein localization. Nevertheless, one should always consider potential side effects of protein tagging and choose a tag that suits protein properties (like size) and experimental conditions. As the perfect tag does not exist, it may be a good idea to perform parallel experiments with all tags to select the best one for a particular application.

In conclusion, our results suggest that our homologous-recombination–based protein tagging strategy using DT40 cells provides a handy tool to study protein localization, abundance and protein–protein interactions as well as polyribosome association in a model vertebrate system.

## SUPPLEMENTARY DATA

Supplementary Data are available at NAR online.

Supplementary Data
