# Supplementary material for: A new strategy for gene targeting and functional proteomics using the DT40 cell line
Source: Nucleic Acids Res. 2013 Jul 27;41(17):e167. doi: 10.1093/nar/gkt650 (PMC3783193; doi:10.1093/nar/gkt650)
Supplement: Supplementary Data [file supp_41_17_e167__index.html]

A new strategy for gene targeting and functional proteomics using the DT40 cell line — A new strategy for gene targeting and functional proteomics using the DT40 cell line — Supplementary Data 

# A new strategy for gene targeting and functional proteomics using the DT40 cell line

## 

files

**Files in this Data Supplement:**

- Supplementary Data - pdf file
- Supplementary Data - xls file
