# Supplementary material for: A new strategy for gene targeting and functional proteomics using the DT40 cell line
Source: Nucleic Acids Res. 2013 Jul 27;41(17):e167. doi: 10.1093/nar/gkt650 (PMC3783193; doi:10.1093/nar/gkt650)
Supplement: Supplementary Data [file supp_gkt650_nar-00934-met-g-2013-File009.pdf]

# Supplementary Data
## accompanying the manuscript:
## "A new strategy for gene targeting and functional proteomics using the DT40 cell line"

by
Kinga P. Orlowska, Kamila Klosowska, Roman J. Szczesny, Dominik Cysewski, Pawel S. Krawczyk and Andrzej Dziembowski

**Supplementary Table S1.** Oligonucleotides used in this study.

**Supplementary Table S2.** Plasmids obtained in this study.

**Supplementary Table S3.** MaxQuant results of CoIP-MS data (.xls file)

**Supplementary Figure S1.** Confirmation of targeted integration of genetic constructs into the DT40 genome.

**Supplementary Figure S2.** Detection of QuantEGFP-tagged proteins in established DT40 cell lines by western blotting using anti-GFP antibodies.

**Supplementary Figure S3.** Results of coimmunoprecipitation using EXOSC8-QuantGFP as bait.

**Supplementary Figure S4.** Results of coimmunoprecipitation using EXOSC9-QuantGFP as bait.

**Supplementary Figure S5.** Results of coimmunoprecipitation using UPF1-QuantGFP as bait.

**Supplementary Figure S6.** Subcellular localization of EXOSC8-EGFP and EXOSC9-EGFP in fixed cells stained with Hoechst33342 and anti-fibrillarin/AlexaFluor555 antibodies.

**Supplementary Table 1.** Oligonucleotides used in this study.

| Primer name | Sequence from 5' to 3' | Use |
|---|---|---|
| ADZ-KO-53 | ACACAATAGAGGCTTGACTGGTGTTCCCAGGGGGCGCTCTTCCGCTTCCTCG | cloning of starting vectors |
| ADZ-KO-55 | CCCCTCGAGTACCGTTCGTATAATGTATGCTATACGAAGTTATTTCCAGCAGGCAGAAGTATG | cloning of starting vectors |
| ADZ-KO-58 | GGGTGAGCTCATGGTGAGCAAGGGCGAGGAG | cloning of starting vectors |
| ADZ-KO-59 | ATCCACTAGTTTACTTGTACAGCTCGTCC | cloning of starting vectors |
| ADZ-KO-60 | CGATTACAAGGATGACGACGATAAGTAGA | cloning of starting vectors |
| ADZ-KO-61 | CTAGTCTACTTATCGTCGTCATCCTTGTAATCGAGCT | cloning of starting vectors |
| ADZ-KO-62 | CGCCAAGCTCTAGCTAGAGGTCGACAAACCATTATTATCATGACATTAACC | cloning of starting vectors |
| ADZ-KO-63 | TGGGGAGGCTTTTTTGGAGGCCTAGGCTTTTGCAAAAAGCTAGCTTGCATG | cloning of starting vectors |
| ADZ-KO-66 | GGCGCTCTTCCGCTTCCTCGCTCACTGACTC | cloning of starting vectors |
| ADZ-KO-77 | TGCAAAGGAAGAGCCTCATGAATTAATTCTTAG | cloning of starting vectors |
| ADZ-KO-93 | GATTACGCCAAGCTCTAGCTAGAGGTCGACCAGACATGATAAGATACATTG | cloning of starting vectors |
| ADZ-KO-94 | CATGCCTGCAGGTCGGCCGCCACGACCGGTGGATTCATTTTTGCAGAAATC | cloning of starting vectors |
| ADZ-KO-1E1 | AAAGCGGCCGCGTCTGTCCAACTTCTTCCTGTG | amplification of homology arms |
| ADZ-KO-1E2 | AAAGCGGCCGCGCTTACCCTGCTTTGATTATG | amplification of homology arms |
| ADZ-KO-1F1 | AAAGCGGCCGCCCTTTAAGGTGAGTTTTTTGG | amplification of homology arms, verification of targeted integration |
| ADZ-KO-1F2 | AAAGCGGCCGCTTAAACCTGTGGAGTTCCCAC | amplification of homology arms |
| ADZ-KO-2E5 | AAAGCGGCCGCTTCTTTACTCACAGGAATGGC | amplification of homology arms |
| ADZ-KO-2E6 | AAAGCGGCCGCTTGCTTAAAGTCAAATGCCCC | amplification of homology arms, verification of targeted integration |
| ADZ-KO-125 | AAAGCGGCCGCCTCCAACGCCTACTGTCACAG | amplification of homology arms |
| ADZ-KO-126 | AAAGCGGCCGCGGGCCACCGTGAATGCAAGAG | amplification of homology arms |
| ADZ-KO-1E3 | ATAGCATACATTATACGAACGGTACTCGAGTGAACAAAAAGATCTCTTCAAAAG | production of inserts for SLIC |
| ADZ-KO-1E4 | GTACAGGCTGGTGATATCGGCGGCAAGCTTCTTGGGCTTTATACTTTTTATTAC | production of inserts for SLIC |
| ADZ-KO-1F3 | ATAGCATACATTATACGAACGGTACTCGAGTAAAGCCATCCCCTTCAATAATG | production of inserts for SLIC |
| ADZ-KO-1F4 | GTACAGGCTGGTGATATCGGCGGCAAGCTTACGAGAAGCTCTCTTCTTTCTC | production of inserts for SLIC |
| ADZ-KO-2E7 | ATAGCATACATTATACGAACGGTACTCGAGTGACATGAAATGAAAGGCCATC | production of inserts for SLIC |
| ADZ-KO-2E8 | GTACAGGCTGGTGATATCGGCGGCAAGCTTTGACTGCTTGTTGGCTTCTTC | production of inserts for SLIC |
| ADZ-KO-127 | ATAGCATACATTATACGAACGGTACTCGAGTAGAGTGTAAGGCACGGGGG | production of inserts for SLIC |
| ADZ-KO-128 | GTACAGGCTGGTGATATCGGCGGCAAGCTTATACTGTGACAGTCCCGTCAC | production of inserts for SLIC |
| ADZ-KO-179 | AAGCTTGAACACTACCTCAGCC | verification of targeted integration |
| ADZ-KO-168 | AACGCCTTGCTGAAACTGACTTC | verification of targeted integration |
| ADZ-KO-2G7 | AGACACTGGATCAGGTTCCAG | verification of targeted integration |
| ADZ-KO-171 | ATTGCAAGAAGTGGCTGAGCAG | verification of targeted integration |
| ADZ-KO-175 | TTCCAAACCCACGACCAGATC | verification of targeted integration |
| ADZ-KO-138 | CATTCTAAGTTTTAATGAGCGGCGC | verification of targeted integration |

**Supplementary Table S2.** Plasmids obtained in this study.

| ID | Name | Addgene ID | Use |
|---|---|---|---|
| pKO1 | pQuantA-hygro-amp | 45584 | basic vectors for cloning of constructs for C-terminal tagging with Quant peptide + protein A |
| pKO2 | pQuantA-hygro-kana | 45585 | |
| pKO3 | pQuantA-puro-kana | 45586 | |
| pKO4 | pQuantA-bsr-kana | 45587 | |
| pKO5 | pQuantEGFP-hygro-kana | 45588 | basic vectors for cloning of constructs for C-terminal tagging with Quant peptide + EGFP |
| pKO6 | pQuantEGFP-puro-kana | 45589 | |
| pKO7 | pQuantEGFP-bsr-kana | 45590 | |
| pKO8 | pQuantFLAG-hygro-kana | 45591 | basic vectors for cloning of constructs for C-terminal tagging with Quant peptide + FLAG |
| pKO9 | pQuantFLAG-puro-kana | 45592 | |
| pKO10 | pQuantFLAG-bsr-kana | 45593 | |
| pKO46 | pEXOSC8-QuantEGFP-puro-kana | - | constructs for C-terminal tagging of EXOSC8 with Quant peptide + EGFP |
| pKO50 | pEXOSC8-QuantEGFP-hygro-kana | - | |
| pKO48 | pEXOSC9-QuantEGFP-puro-kana | - | constructs for C-terminal tagging of EXOSC9 with Quant peptide + EGFP |
| pKO59 | pEXOSC9-QuantEGFP-hygro-kana | - | |
| pKO52 | pCNOT7-QuantEGFP-puro-kana | - | constructs for C-terminal tagging of CNOT7 with Quant peptide + EGFP |
| pKO66 | pCNOT7-QuantEGFP-hygro-kana | - | |
| pADZ450 | pUPF1-QuantEGFP-puro-kana | - | constructs for C-terminal tagging of UPF1 with Quant peptide + EGFP |
| pADZ525 | pUPF1-QuantEGFP-hygro-kana | - | |

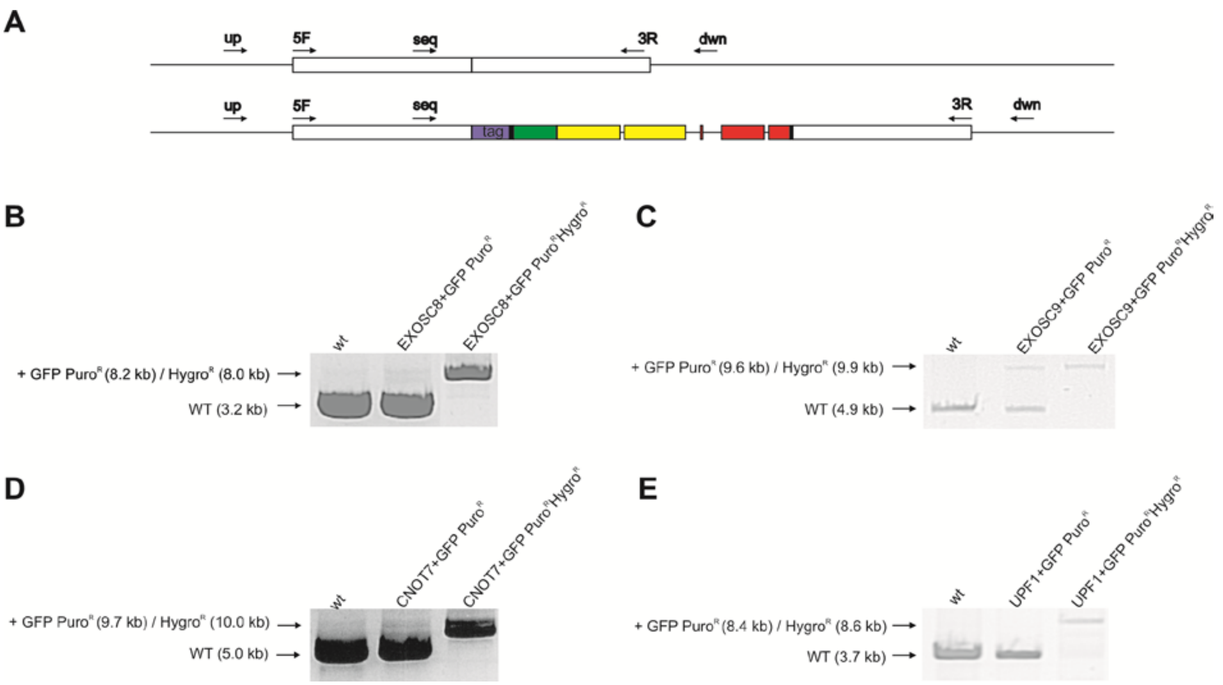


**Supplementary Figure S1.** Confirmation of targeted integration of genetic constructs into the DT40 genome. A) Schematic view of gene-specific primers used for verification, B) Confirmation of C-terminal tagging of EXOSC8 by PCR using seq and dwn primers (ADZ-KO-179, ADZ-KO-2G7), C) Confirmation of C-terminal tagging of EXOSC9 by PCR using 5F and dwn primers (ADZ-KO-1F1, ADZ-KO-168), D) Confirmation of C-terminal tagging of CNOT7 by PCR using up and 3R primers (ADZ-KO-171, ADZ-KO-2E6), E) Confirmation of C-terminal tagging of UPF1 by PCR using seq and dwn primers (ADZ-KO-175 and ADZ-KO-138). Preference for wt PCR product can be observed in the heterozygote for most genes.

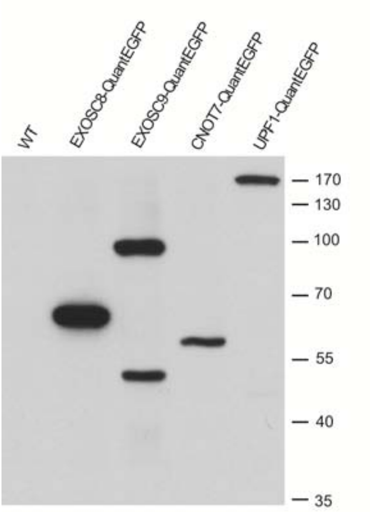


**Supplementary Figure S2.** Detection of QuantEGFP-tagged proteins in established DT40 cell lines by western blotting using anti-GFP antibodies. In the case of EXOSC9, an additional band is visible due to specific protein degradation.

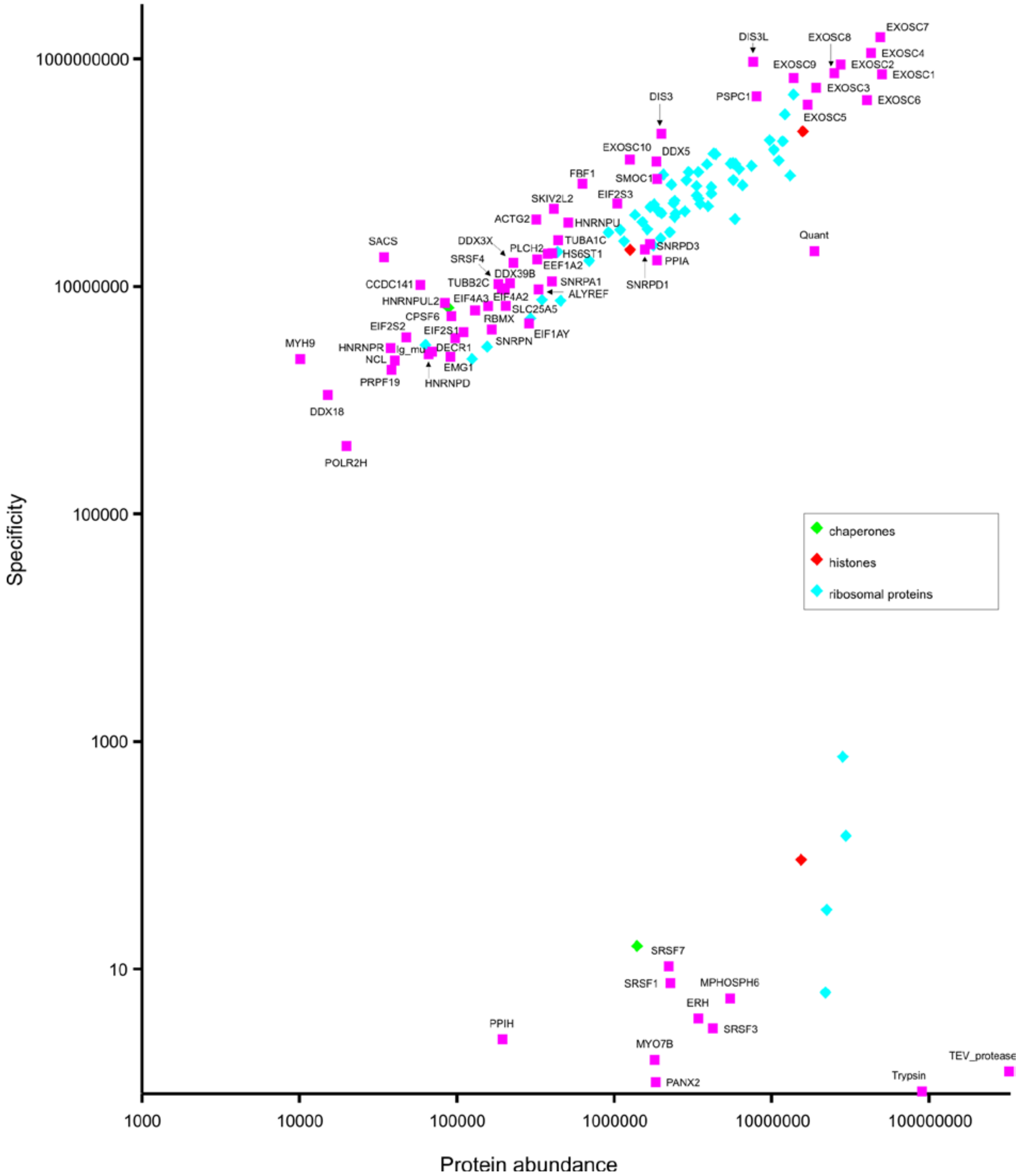


**Supplementary Figure S3.** Results of coimmunoprecipitation using EXOSC8-QuantGFP as bait.

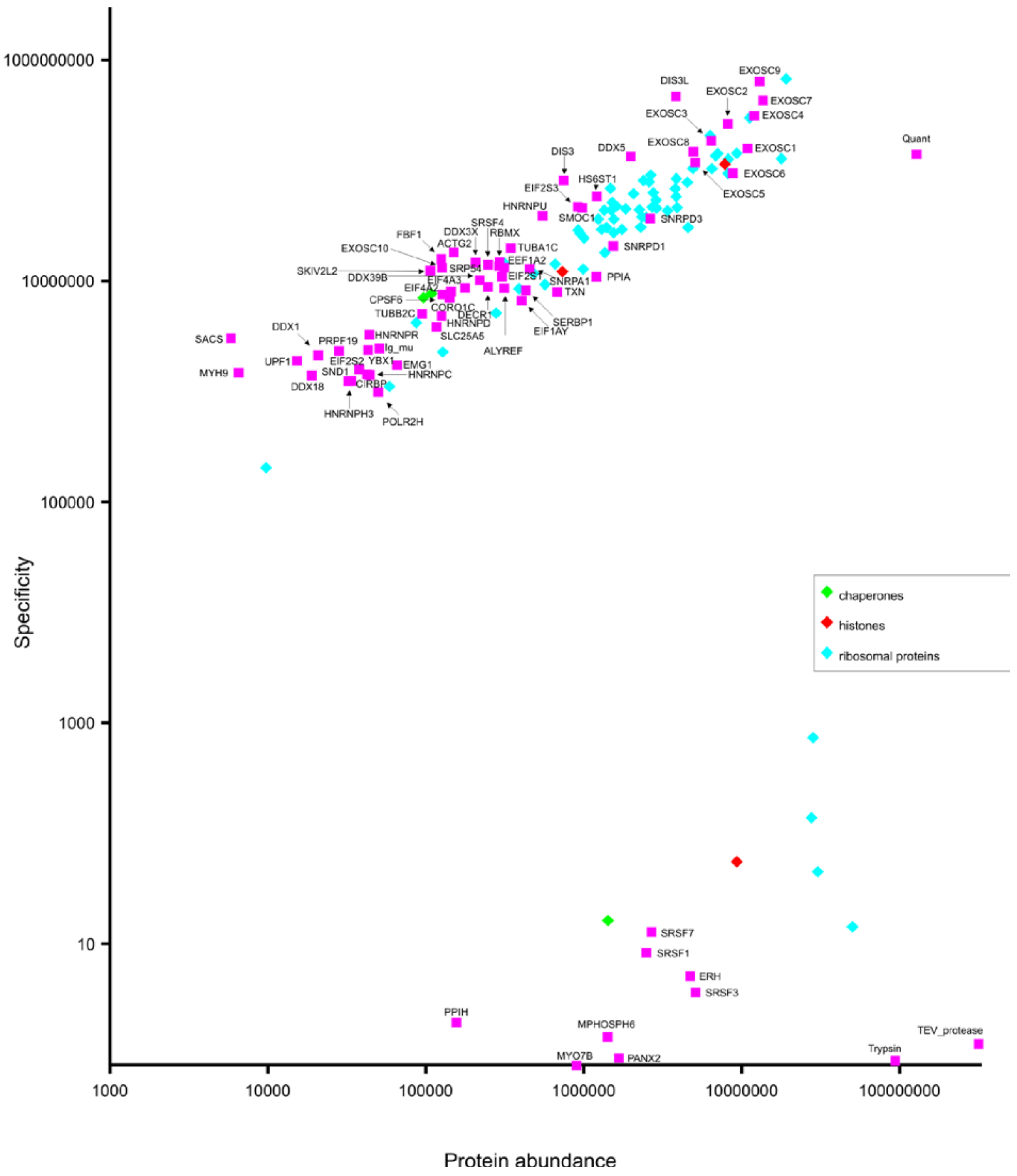


**Supplementary Figure S4.** Results of coimmunoprecipitation using EXOSC9-QuantGFP as bait.

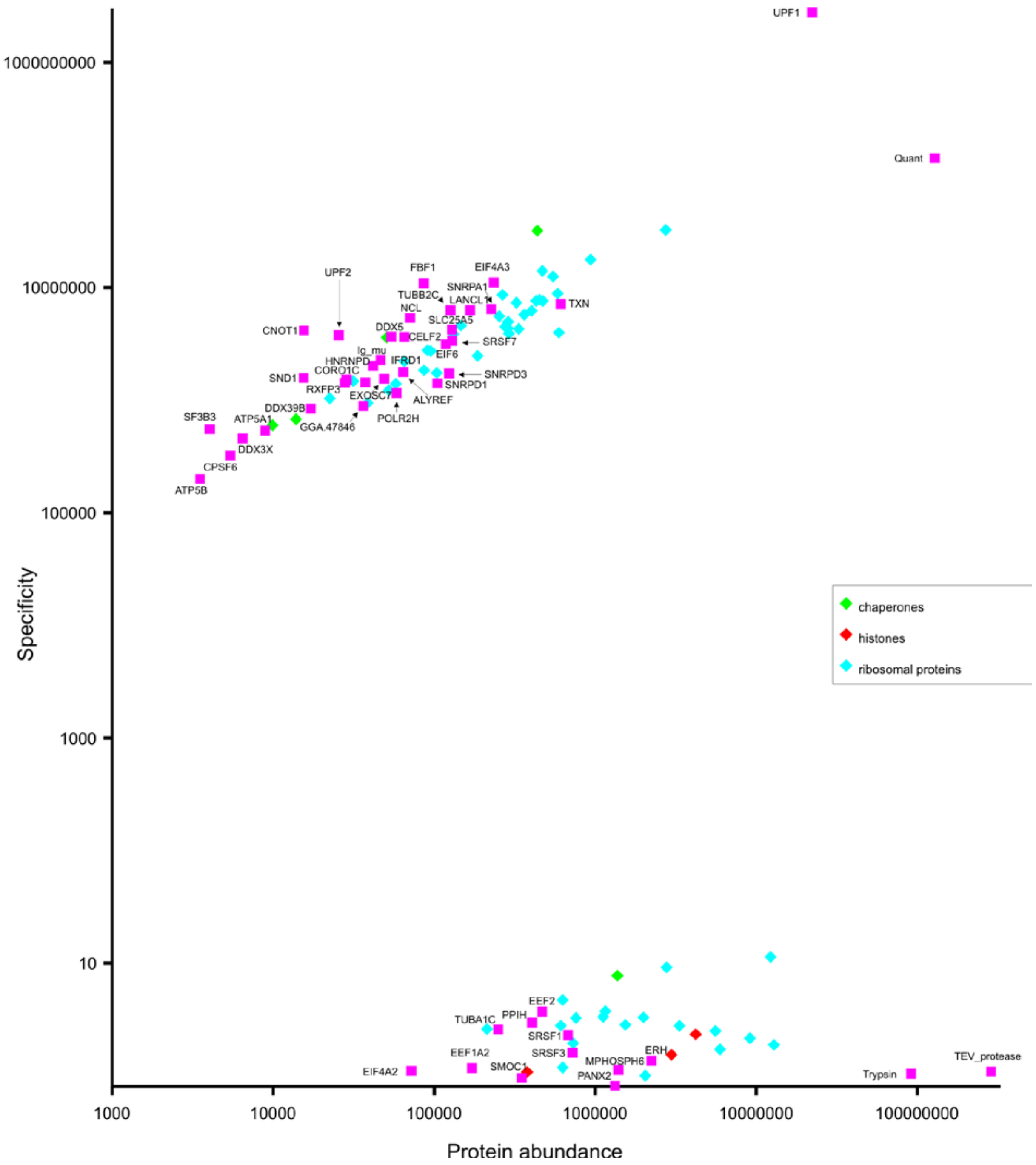


**Supplementary Figure S5.** Results of coimmunoprecipitation using UPF1-QuantGFP as bait.

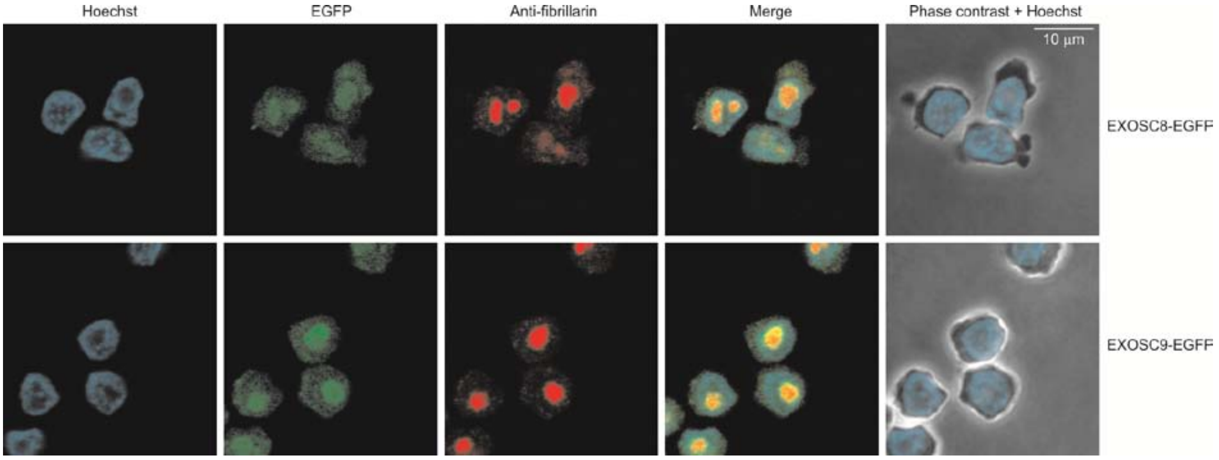


**Supplementary Figure S6.** Subcellular localization of EXOSC8-EGFP and EXOSC9-EGFP in fixed cells stained with Hoechst33342 and anti-fibrillarin/AlexaFluor555 antibodies. Enrichment in the nucleoli can be seen for both proteins.
